# Factors associated with syphilis infection: a cross-sectional survey among outpatients in Asikuma Odoben Brakwa District, Ghana

**DOI:** 10.1186/s12879-019-3967-6

**Published:** 2019-04-29

**Authors:** Martin Banong-le, Samuel Kwabena Ofosu, Francis Anto

**Affiliations:** 1 School of Public Health, University of Ghana, Legon, Accra, Ghana; 2 Ministry of Health, College of Nursing and Midwifery, Nalerigu, Northern Region Ghana; 3 District Health Directorate, Ghana Health Service, Breman Asikuma, Central Region Ghana

**Keywords:** *Treponema pallidum*, Syphilis, Sexually transmitted infection, Coerced sexual intercourse

## Abstract

**Background:**

Syphilis is a sexually transmitted infection caused by the bacterium *Treponema pallidum.* The disease affects all ages and both sexes but more prevalent among the sexually active age group of 15–49 years. The purpose of the current study was to determine the prevalence and factors associated with syphilis infection among outpatients 15–49 years in the Asikuma Odoben Brakwa District of Ghana where high levels of infection were earlier reported among antenatal women.

**Methods:**

A descriptive cross-sectional study was carried out in 13 randomly selected health facilities. Blood samples were collected and tested for syphilis infection and a questionnaire administered to determine factors associated with the disease.

**Results:**

A total 277 patients aged 15–49 years participated in the study. The overall prevalence of syphilis infection was 3.2% (9/277), with 5.7% (6/105) and 1.7% (3/172) among males and females respectively. Significant factors associated with syphilis infection included sub-district of residence, (χ^2^ (4) = 31.20, *p* < 0.001) and history of coerced sexual intercourse (χ^2^ (1) =7.49, *p* = 0.006).

**Conclusions:**

The prevalence of syphilis infection was high among male patients who lived in rural areas. Having a history of coerced sexual intercourse was a strong predictor for syphilis infection. Access to sexually transmitted infection control interventions in rural communities including health education may help control the disease.

**Electronic supplementary material:**

The online version of this article (10.1186/s12879-019-3967-6) contains supplementary material, which is available to authorized users.

## Background

Syphilis is a sexually transmitted infection that remains a major global public health problem [1]. The disease is caused by the bacterium *Treponema pallidum* (a spirochete) and is one of the four most common curable sexually transmitted infections. Syphilis affects all age groups and both sexes, but most prevalent among the most sexually active age group of 15–49 years.

The disease can be transmitted by a pregnant woman to her unborn baby in utero [2], leading to neonatal death, blindness in the newborn and severe disability in infants [3]. Major risk factors that promote the transmission of syphilis include men having sex with men, having concurrent multiple sex partners, alcohol or drug use and engaging in unprotected sex [4].

Even though an effective treatment for syphilis has been available since the 1940s, over 10 million people still acquire the disease worldwide every year with more than 90% of these new cases occurring in developing countries [5].

In Africa, the estimated prevalence of syphilis was 0.2% in 2012 [3]. It was also reported that syphilis prevalence among the general population in Rwanda was 0.9% [1] and 1.8% in Kenya [6]. The prevalence among blood donors ranges from 1.5% in Burkina Faso, 3.7% in Congo, 8.4% in South Africa to 17.4% in Cameroon [7, 8]. Prevalence of the disease among patients in the Cape Coast metropolis of Ghana was 8.5% [9]. Other major cities in Ghana have recorded 7.9% (Accra) [10] and 4.5% (Kumasi) [11] prevalence among the general population.

Available records from the Asikuma Odoben Brakwa District indicate a prevalence of 30.5% in 2008 [12] and 1.6% in 2016 [13] among pregnant women attending health facilities. The Central region of Ghana including the Asikuma Odoben Brakwa District is endemic for yaws [14] which is another treponemal infection. Over the past five years however, no case of yaws has been reported from the district. The prevalence of syphilis in such areas is reported to be overestimated when a test algorithm that involves two treponemal tests is used because of the inability of the tests to classify and distinguish active syphilis infection from previous infection or other treponemal infections including yaws. To address this challenge, a test algorithm involving a non-treponemal test such as Rapid Plasma Reagin (RPR) followed by a treponemal test was recommended [15].

In Ghana, emphasis is placed on screening pregnant women for syphilis during antenatal care to prevent mother-to-child transmission. This population group however, forms only about 4.0% of the total population. The prevalence of the disease in women who are not pregnant but are sexually active and their male sex partners is also very important in the prevention and control of sexually transmitted infection. This study therefore determined the prevalence and factors associated with syphilis infection among outpatients aged 15–49 years seeking general medical care at the Asikuma Odoben Brakwa District in Ghana.

## Methods

### Study site

The study was conducted in the Asikuma-Odoben-Brakwa District in the Central Region of Ghana. The district is mainly rural with a population of about 131,000, living in 245 settlements and engaged mainly in agriculture. For health administrative purposes, the district has been divided into four sub-districts namely Asikuma, Odoben, Brakwa and Anhwiam. The district has only one hospital which serves as a referral centre, three health centres, 20 health compounds for the delivery of community-based health services and one maternity home.

### Study design

A health facility-based cross-sectional survey was carried out in the Asikuma-Odoben-Brakwa District from October, 2016 to July, 2017. The health facilities included the district hospital, three health centres and nine Community-based Health Planning and Services (CHPS) compounds. Patients aged 15–49 years who sought outpatient services in these facilities were eligible to participate in the study. Those referred to the laboratory for blood-based tests were screened and their demographic and behavioural data collected using a case record form and blood samples collected for laboratory analysis.

### Sample size estimation and sampling

The sample size was estimated using the Cochran, 1977 formula n = z^2^pq/d^2^ [16]. Where, z is the critical value corresponding to 95% confidence level (1.96), p is the proportion of syphilis reported in the district = 1.6% or 0.016 [13], q the power (1-p, =1–0.016 = 0.984) and d is the margin of error (0.05). The minimum sample size (n) required for this study was 25. Making room for 10% non-response, a sample of 27 was deemed appropriate.

The three health centres and the district hospital were purposively included as study facilities. Nine of the CHPS compounds were randomly selected out of the twenty in the district. The sample size was proportionately distributed among the 13 selected health facilities based on the 2016 Out Patient attendance. Participants were consecutively enrolled into the study as they were referred by the attending clinician to the laboratory for blood-based investigations.

### Data collection

Data on demographic characteristics of participants and factors likely to be associated with syphilis infection were collected directly from the study participants through face to face interview and recorded on a structured questionnaire developed specifically for this study. These data included, the educational level, alcohol consumption, number of sex partners, marital status, history of sexually transmitted infection, history of blood transfusion and age at first sexual encounter. Finger prick or venous blood samples were collected and used to perform Rapid First Response Syphilis Anti-TP Card Test. A confirmatory Treponema Pallidum Haemagglutination Assay (TPHA) test was performed on samples that were found reactive to the First Response Syphilis Anti-TP Card test.

### Sample collection and test procedure for first response syphilis anti-TP test

At the health centres and CHPS compounds finger prick blood samples were collected and used for First Response Syphilis Anti-TP Test as these facilities are not staffed with trained phlebotomists. The finger tip of the patient was cleaned with an alcohol swab and allowed to air dry. Blood samples were obtained using sterile lancet finger prick and use of a sample pipette. The First Response Syphilis Anti-TP Card Test kit components were brought to room temperature. The test device was removed from the foil pouch and placed on a flat, dry surface. One drop (about 20 μl) of whole blood in the sample pipette was added to the sample well in the test kit followed by 3 drops (about 70 μl) of assay diluent to the sample well. This was observed for development of coloured bands in the result window after 20 min and interpreted as either reactive or nonreactive. Participants who were reactive to the First Response Syphilis Anti-TP Card Test were invited to the district hospital for the confirmatory TPHA test.

### Specimen collection and preparation for TPHA test

At the district hospital, 5 ml of venous blood was collected from each patient by qualified laboratory staff. About 200 μl of blood was added to labelled ethylenediaminetetraacetic acid (EDTA) test tubes to prevent coagulation. The blood samples were centrifuged and the plasma collected and frozen at − 15 °C. One microliter of blood sample was then added into the test tube to obtain sera that was used for TPHA confirmation test.

### Test procedure for TPHA test

Three wells of a microtitration plate were allowed to reach room temperature and 190 μl of diluent added to well one followed by 10 μl of serum. A micropipette was used to mix the contents of well one, after which 25 μl of the mixture was transferred to well two and well three. Contents were shaken gently and test and control cells were re-suspended. Seventy five microliters of control cells were added to wells two and three, shaken and contents allowed to mix thoroughly. The plate was covered and protected from direct sunlight, heat and any source of vibration and then were incubated for 45 to 60 min at room temperature. The results were read and interpreted as positive or negative for syphilis.

### Data management and analysis

Data collected were checked for completeness and accuracy. Codes on questionnaires were cross-checked with those on the laboratory test forms to ensure consistency. Data entry was done in Excel version 2013 and imported into Stata version 14 for statistical analysis. Frequencies were computed on the syphilis test results as either positive or negative and proportions determined to measure the prevalence of syphilis infection. Chi-square test of association with 5% significance level was used to determine factors associated with syphilis infection. Factors that were statistically significant (*p* = 0.05) in the chi-square test were assessed using logistic regression to determine the strength of association with the dependent variable (syphilis infection).

## Results

### Demographic characteristics of participants

A total of 277 patients (62.1%, 172/277 females), aged 15–49 years (mean: 29.02, SD: 8.72), mainly single (48.4%), public servants (26.4%) and farmers (20.9%) participated in the study. Majority of the study participants were Christians (90.3%), with basic level education (50.1%) and reside mainly in rural communities. Thirty (10.8%) of the respondents reported that they have never had sex before (Table 1).

**Table 1:** Demographic Characteristics of Respondents

Demographic characteristics (*N* = 277)		Number	Percentage
Age group (yrs.)	15–19	38	13.7
	20–24	60	21.7
	25–29	64	23.1
	30–34	40	14.4
	35–39	32	11.6
	40–44	19	6.9
	45–49	24	8.7
Sex	Male	105	37.9
	Female	172	62.1
Marital status	Single	134	48.4
	Married	122	44.0
	Divorced/separated	21	7.6
Sexual history	Ever had sex	247	89.2
	Never had sex	30	10.8
Occupation	Farming	58	20.9
	Trading	70	25.3
	Artisan	16	5.8
	Public servant	73	26.4
	Student	55	19.9
	Seamstress	5	1.8
Religion	Christian	250	90.3
	Muslim	19	6.9
	Traditionalist	8	2.9
Educational level	No formal education	14	5.1
	Primary	140	50.1
	Secondary	63	22.7
	Tertiary	60	21.7
Residence	Rural	191	68.9
	Urban	86	31.1

### Prevalence of syphilis infection

Of the 277 outpatients tested, nine of them were reactive to First Response Syphilis Anti-TP Test. All the nine (100%) reactive samples were positive for the TPHA confirmatory test hence, the overall prevalence of syphilis using both First Response Syphilis Anti-TP Test and TPHA confirmatory test was 3.2% (9/277), (95% CI, 1.7–6.1). Infection was found in all age groups with those aged ≥40 years (7.0%, 3/43) and 15–19 years (5.3%, 2/38) being the most infected. More males (5.7%, 6/105) than females (1.7%, 3/172) were infected. Seven patients out of the 191 (3.7%) participants from the rural communities were infected with 21.7% (5/23) of those resident in the Anhwiam sub-district being infected (Table 2).

**Table 2:** Distribution of syphilis prevalence by demographic characteristics

Demographic Characteristics		N (%)	Syphilis test (TPHA test)	
			Positive (%)	Negative (%)
Age group	Overall (15–49)	277 (100)	9 (3.2)	268 (96.8)
	15–19	38 (13.7)	2 (5.3)	36 (94.7)
	20–24	60 (21.7)	1 (1.7)	59 (98.3)
	25–29	64 (23.1)	1 (1.6)	63 (98.4)
	30–34	40 (14.4)	1 (2.5)	39 (97.5)
	35–39	32 (11.6)	1 (3.1)	31 (96.9)
	40 and above	43 (15.5)	3 (7.0)	40 (93.0)
Sex	Male	105 (37.9)	6 (5.7)	99 (94.3)
	Female	172 (62.1)	3 (1.7)	169 (98.3)
Marital status	Single	134 (48.4)	4 (3.0)	130 (97.0)
	Married	122 (44.0)	5 (4.1)	117 (95.9)
	Divorced/separated	21 (7.6)	0 (0.0)	21 (100.0)
Occupation	Farming	58 (20.9)	2 (3.4)	56 (96.6)
	Trading	70 (25.3)	1 (1.4)	69 (98.6)
	Artisan	16 (5.8)	1 (6.3)	15 (93.7)
	Public servant	73 (26.4)	2 (2.7)	71 (97.3)
	Student	55 (19.9)	3 (5.5)	52 (94.5)
	Seamstress	5 (1.8)	0 (0.0)	5 (100.0)
Religion	Christian	250 (90.2)	8 (3.2)	242 (96.7)
	Muslim	19 (6.9)	1 (5.3)	18 (94.7)
	Traditionalist	8 (2.9)	0 (0.0)	8 (100.0)
Education	No formal education	14 (5.1)	0 (0.0)	14 (100.0)
	Primary	140 (50.5)	4 (2.9)	136 (97.1)
	Secondary	63 (22.7)	2 (3.2)	61 (96.8)
	Tertiary	60 (21.7)	3 (5.0)	57 (95.0)
Residence	Rural	191 (68.9)	7 (3.7)	184 (96.3)
	Urban	86 (31.1)	2 (2.3)	84 (97.7)
Subdistrict	Asikuma	102 (36.8)	1 (1.0)	101 (99.0)
	Odoben	51 (18.4)	3 (5.9)	48 (94.1)
	Brakwa	64 (23.1)	0 (0.0)	64 (100.0
	Anhwiam	23 (8.3)	5 (21.7)	18 (78.3)
	Outside district	37 (13.4)	0 (0.0)	37 (100.0)

### Factors associated with syphilis infection

Chi-square test was performed to assess the association between demographic factors, sexual behaviour profiles of participants and syphilis infection. Although more males (5.7%) than females (1.7%) were found to be infected, the difference was not statistically significant (χ^2^ (1) = 3.27, *p* = 0.071). Similarly, syphilis infection was more prevalent among patients who were married (4.1%) compared to those who were single (3.0%), however, the difference was not statistically significant (χ^2^ (2) = 0.01, *p* = 0.602). Statistically significant association was however found between sub-district of residence and syphilis infection (χ^2^ (4) = 31.20, *p* < 0.001). Compared with the other sub-districts, Anhwiam sub-district had the highest (21.7%) prevalence of infection, followed by Odoben sub-district (5.9%). No statistically significant association was found between religion (χ^2^ (2) = 0.52, *p* = 0.773), educational level (χ^2^ (3) = 1.12, *p* = 0.771), condom use (χ^2^ (1) = 0.21, *p* = 0.648), and syphilis infection (Table 3a).

**Table 3:** Factors Associated with Syphilis Infection

Characteristic	N (%)	Syphilis test		X^2^(df)	*p*-value
		Positive (%)	Negative (%)		
Sex				3.27(1)	0.071
Male	105 (37.9)	6 (5.7)	99 (94.3)		
Female	172 (62.1)	3 (1.7)	169 (98.3)		
Marital status				1.01(2)	0.602
Single	134 (48.4)	4 (3.0)	130 (97.0)		
Married	122 (44.0)	5 (4.1)	117 (95.9)		
Divorced/separated	21 (7.6)	0 (0.0)	21 (100.0)		
Subdistrict				31.20(4)	< 0.001
Asikuma	102 (36.8)	1 (1.0)	101 (99.0)		
Odoben	51 (18.4)	3 (5.9)	48 (94.1)		
Brakwa	64 (23.1)	0 (0.0)	64 (100.0		
Anhwiam	23 (8.3)	5 (21.7)	18 (78.3)		
Outside district	37 (13.4)	0 (0.0)	37 (100.0)		
Religion				0.52(2)	0.773
Christian	250 (90.2)	8 (3.2)	242 (96.7)		
Muslim	19 (6.9)	1 (5.3)	18 (94.7)		
Traditionalist	8 (2.9)	0 (0.0)	8 (100.0)		
Education				1.12(3)	0.771
No formal education	14 (5.1)	0 (0.0)	14 (100.0)		
Primary	140 (50.5)	4 (2.9)	136 (97.1)		
Secondary	63 (22.7)	2 (3.2)	61 (96.8)		
Tertiary	60 (21.7)	3 (5.0)	57 (95.0)		
Condom use at first sexual intercourse				0.21(1)	0.648
Used condom	6 (26.7)	3 (4.5)	63 (95.5)		
Did not use condom	181 (73.3)	6 (3.3)	175 (96.7)		
Frequency of condom use				5.13(4)	0.275
Always	12 (4.9)	1 (8.3)	11 (91.7)		
Very often	23 (9.3)	0 (0.0)	23 (100.0)		
Rarely	40 (16.2)	0 (0.0)	40 (100.0)		
Very rarely	51 (20.7)	1 (2.0)	50 (98.0)		
Never used one	121 (48.9)	7 (5.8)	114 (94.2)		
History of coerced sex				7.49(1)	0.006
Ever had coerced sex	49 (19.8)	5 (10.2)	44 (89.8)		
Never had coerced sex	198 (80.2)	4 (2.0)	194 (98.0)		
Number of sex partners in the past year				3.83(2)	0.147
None	28 (11.34)	1 (3.6)	27 (96.4)		
One	150 (60.73)	8 (5.3)	142 (94.7)		
More than one	69 (27.94)	0 (0.0)	69 (100.0)		
Casual sex in the past year				2.49(1)	0.115
Had casual sex	52 (21.1)	0 (0.0)	52 (100.0)		
Did not have casual sex	195 (78.9)	9 (4.6)	186 (95.4)		
Sex in exchange for money or gifts				1.36(1)	0.244
Had	26 (10.5)	2 (7.7)	24 (92.3)		
Did not have	221 (89.5)	7 (3.5)	214 (96.8)		
History of blood transfusion				0.17(2)	0.916
Blood transfused	32 (11.6)	1 (3.1)	31 (96.9)		
No blood transfusion	240 (86.6)	8 (3.3)	232 (96.7)		
Do not know	5 (1.8)	0 (0.0)	5 (100.0)		

There was a high prevalence (10.2%) of syphilis infection among respondents who reported a history of coerced sexual intercourse (χ^2^ (2) =7.49, *p* = 0.006). No infection was observed among respondents who indicated they had more than one sex partner in the past year compared with 1.8% prevalence among respondents who reported having had only one sex partner within the past year. This association was however, insignificant (χ^2^ (2) = 3.83, *p* = 0.147). No statistically significant association was found between having casual sex within the past year (χ^2^ (1) = 2.49, *p* = 0.115), having sex in exchange for money or gifts (χ^2^ (1) = 1.36, *p* = 0.244), history of blood transfusion (χ^2^ (2) = 0.17, *p* = 0.916) and syphilis infection (Table 3b).

### Bivariate and multivariate analyses of factors associated with syphilis infection

TPHA test result was considered the outcome variable and sub-district andhistory of coerced sex were used as the independent variables in the logistic regression model. The outcome of the bivariate and the multivariate analyses are presented in Table 4.

**Table 4:** Factors Associated with Syphilis Infection (Crude and Adjusted Odds Ratios)

Characteristic	Crude Odds Ratio (OR)			Adjusted Odds Ratio (aOR)		
	OR	95% CI	*p*-value	aOR	95% CI	*p*-value
Subdistrict						
Asikuma	1.00			1.00		
Odoben	6.31	0.64–62.28	0.115	4.81	1.43–49.85	0.041
Anhwiam	28.06	3.09–254.42	0.003	30.85	3.08–308.65	0.004
History of coerced sex						
Coerced sex	1.00			1.00		
No coerced sex	0.16	0.04–0.63	0.008	0.12	0.02–0.61	0.010

In the bivariate model, the odds of a patient having a positive test result for syphilis infection was 6.31 times if that patient lived in Odoben sub-district compared with patients who lived in Asikuma sub-district (OR = 6.31 [95% CI = 0.64–62.28], *p* = 0.115). Also, the odds of having a positive test result for syphilis infection was 28.06 times in patients from Anhwiam sub-district compared with patients from Asikuma sub-district (OR = 28.06 [95% CI = 3.09–254.42], *p* = 0.003). It was observed that the odds of having a positive test results for syphilis infection was 0.16 times lower in patients who reported having had no history of coerced sexual intercourse compared with patients who reported they had ever experienced coerced sexual intercourse (OR = 0.16 [95% CI = 0.04–0.63], *p* = 0.008).

In the multivariate model, the odds of a patient having a positive test result for syphilis infection was 4.81 times higher if the patient lived in Odoben sub-district compared with patients who lived in Asikuma sub-district (aOR = 4.81 [95% CI = 1.43–49.85], *p* = 0.041), and 30.85 times higher in patients who lived in Anhwiam sub-district compared with those who lived in Asikuma sub-district (aOR =30.85 [95% CI = 3.08–308.65], *p* = 0.004), adjusting for history of coerced sex. Adjusting for sub-district of patient, the odds of having a positive test results for syphilis infection was 0.12 times lower in patients who reported having had no history of coerced sexual intercourse compared with patients who reported a history of coerced sexual intercourse (aOR = 0.12 [95% CI = 0.02–0.61], *p* = 0.010).

## Discussion

The study revealed an overall syphilis infection prevalence of 3.2%, with 5.7% prevalence among males and 1.7% among females. A strong association was found between syphilis infection and two main factors. These are: being resident in the Anhwiam sub-district and having a history of coerced sexual intercourse.

The overall prevalence of syphilis infection in the current study falls within ranges reported from various parts of sub-Sahara Africa and among different population groups. In a hospital-based study among pregnant women and blood donors in Cameroon, Dionne-Odom and colleagues found prevalences ranging from 1.3 to 3.8% [17]. Similarly, a prevalence of 2.9% was reported in a cross-sectional hospital-based survey among pregnant women in Northwest Ethiopia [18]. Other studies have reported 2.0 to 3.8% from Rwanda [19] and 5.1% from South Ethiopia [20].

In an earlier study in northern Ghana by Kubio et al. [21], a prevalence of 4.7% was reported whilst Asiki, et al. [22], found 4.3% prevalence in Uganda. The prevalence level found in the current study is much lower than the 9.3% reported by Sakala et al. [23] from Zambia and 7.3% by Shimelis and colleagues from Ethiopia [24]. Though the level of infection detected in the Asikuma Odoben Brakwa District, in Ghana appears to be low, it should be taken seriously as persons with syphilis infection are more likely to have co-morbid sexually transmitted infections including HIV [19, 20, 25], which were not screened for in the current study.

Much lower prevalences have been reported from some other African countries, including the 0.9, 1.4 and 2.2% reported from Rwanda, Swaziland and South Africa respectively [1, 26, 27]. Similarly, the infection level in the current study was higher compared with the 1.6% reported from Human Immunodeficiency Virus (HIV) sentinel survey among pregnant women seeking antenatal care (ANC) in the Asikuma Odoben Brakwa District in 2016 [13].

Several factors including age, area of residence, level of formal education [19], history of miscarriage and stillbirth [18] have been reported to be associated with syphilis infection. Other factors include having a history of blood transfusion and being male [24]. Similar to the findings from the current study, earlier studies reported from Ethiopia found more males than females with syphilis infection [24, 26]. Though the difference found in this study was not statistically significant, it is worthy of note when it comes to public health interventions. A review of records on screened blood samples collected from prospective blood donors in the Ho municipal hospital in Ghana also revealed a slightly higher level of syphilis infection among the male donors compared to the females [27]. According to Urassa and colleagues, [28] males usually have more sexual partners, as they desire to gain social status and be respected as men among their male peers.

The sub-district of residence was found to be a strong predictor for syphilis infection in the current study. Participants who lived in Anhwiam sub-district were more likely to have positive test results for syphilis infection compared to those resident in Asikuma sub-district similar to reports from the HIV sentinel sites survey [13]. This could be due to geographical differences between the sub-districts as Anhwiam sub-district for example is very remote with only two CHPS compounds (the very basic health service unit in Ghana). Communities in that sub-district lack access to commodities like condoms for prevention of STIs as well as effective health education programmes. In a similar cross-sectional study among antenatal women in Northern Tanzania, syphilis prevalence was found to be higher in rural clinics than those in the urban area [29]. Significant association has been reported between history of coerced sex and syphilis infection in earlier studies. Coerced sex may lead to physical trauma to the genitalia during intercourse that may facilitate the transmission of syphilis. According to Nankinga et al., there is a strong association between sexual violence, the number of lifetime partners [30], economic deprivation and acquisition of sexually transmitted infections [31]. Intimate partner violence has been linked to poor relationships and lack of self-control, which are often accompanied by extramarital relations.

## Limitations

The study had some limitations. First, because of the testing algorithm used (First Response Syphilis Anti-TP Card test followed by TPHA), which are both treponemal tests, the study could not distinguish active syphilis infection from previous infections or non-venereal treponemal infections such as yaws. This could contribute to overestimation of the prevalence of the disease in the district [15]. Ghana is known to be endemic for yaws which primarily affects children aged under 15 years living in rural communities. There has however, been substantial decline in reported cases since 2009 [32]. Current evidence also show that some clinically suspected yaws cases are due to infection by *Haemophilus ducreyi* [33–35] with most of these lesions remaining unexplained. Available data from the district (District Health Information Management System 2-DHIMS2) show that from 2014 to 2018, there was no reported case of yaws from the district (Additional file 1). However, over the same period, there have been reported cases of genital ulcers among both males and females with 49 cases in 2014, 20 in 2015 and five in 2018 (Additional file 1). The level of overestimation (if any) as a result of our test algorithm is likely to be very minimal. Second, patients who reported to health facilities to seek medical attention and were required to do other laboratory investigations were those that were selected for the study excluding those who were not referred for laboratory investigations. It is therefore possible that some outpatients who were not included in the study because they did not meet the inclusion criteria could be positive for syphilis infection. This could affect the estimation of the true prevalence among outpatient attendees and so the results should be interpreted with some level of caution. These limitations notwithstanding, the findings of this study can serve as a valuable guide in the design of interventions aimed at controlling syphilis in the district.

## Conclusion

The overall syphilis prevalence was 3.2%, with a higher level of infection among males than females. Being resident in the Anhwiam subdistrict and engaging in coerced sexual intercourse were found to be strong predictors for syphilis infection. The prevalence level detected in the current study was higher than the 1.6% reported among pregnant women, through the HIV sentinel survey in 2016. We recommend health interventions that will target all age groups, especially, those in the rural communities.

## Additional file

Additional file 1: Syphilis Prevalence Study-AOBD. Raw data of Syphilis Prevalence Study-AOBD. (XLSX 52 kb)

## Abbreviations

ANC: Antenatal care
CHPS: Community-based health planning and services
DHIMS2: District health information management system 2
EDTA: Ethylenediaminetetraacetic acid
HIV: Human immunodeficiency virus
RPR: Rapid plasma reagin
TPHA: Treponema palladium haemagglutination assay

## References

[1] Mutagoma Mwumvaneza, Remera Eric, Sebuhoro Dieudonné, Kanters Steve, Riedel David J., Nsanzimana Sabin (2016). The Prevalence of Syphilis Infection and Its Associated Factors in the General Population of Rwanda: A National Household-Based Survey. Journal of Sexually Transmitted Diseases.

[2] Kamb Mary L., Newman Lori M., Riley Patricia L., Mark Jennifer, Hawkes Sarah J., Malik Tasneem, Broutet Nathalie (2010). A Road Map for the Global Elimination of Congenital Syphilis. Obstetrics and Gynecology International.

[3] Newman Lori, Rowley Jane, Vander Hoorn Stephen, Wijesooriya Nalinka Saman, Unemo Magnus, Low Nicola, Stevens Gretchen, Gottlieb Sami, Kiarie James, Temmerman Marleen (2015). Global Estimates of the Prevalence and Incidence of Four Curable Sexually Transmitted Infections in 2012 Based on Systematic Review and Global Reporting. PLOS ONE.

[4] Park Hayoung, Konda Kelika A., Roberts Chelsea P., Maguiña Jorge L., Leon Segundo R., Clark Jesse L., Coates Thomas J., Caceres Carlos F., Klausner Jeffrey D. (2016). Risk Factors Associated with Incident Syphilis in a Cohort of High-Risk Men in Peru. PLOS ONE.

[5] Trope Lee A, Wijesooriya Nalinka Saman, Broutet Nathalie, Temmerman Marleen, Newman Lori (2014). Reaching beyond pregnant women to eliminate mother-to-child transmission of syphilis in Africa. Expert Review of Anti-infective Therapy.

[6] Otieno-Nyunya B, Bennett E, Bunnell R, Dadabhai S, Gichangi AA, Mugo N, Wanyungu J, Baya I, Kaiser R (2011). Epidemiology of syphilis in Kenya: results from a nationally representative serological survey. Sex Transm Infect.

[7] Batina A, Kabemba S, Malengela R (2007). Infectious markers among blood donors in Democratic Republic of Congo (DRC). Rev Med Brux.

[8] Bisseye C, Sanou M, Nagalo BM, Kiba A, Compaore TR, Tao I, Simpore J (2013). Epidemiology of syphilis in regional blood transfusion centres in Burkina Faso, West Africa. Pan Afr Med J..

[9] Faustina Nti-Boakye, Dankwa Kwabena, Ampiah Charles, Boampong Johnson, Nuvor Samuel (2015). Seroprevalence of Syphilis Infection in Individuals at Cape Coast Metropolis, Ghana. British Journal of Medicine and Medical Research.

[10] Adjei AA, Kudzi W, Armah H, Adiku T, Amoah AG, Ansah J (2003). Prevalence of antibodies to syphilis among blood donors in Accra, Ghana. Jpn J Infect Dis.

[11] Owusu-Ofori AK, Parry CM, Bates I (2011). Transfusion-transmitted syphilis in teaching hospital, Ghana. Emerg Infect Dis.

[12] National AIDS/STI Control Programme/Ghana Health Service. HIV sentinel survey report 2008. Accra: National AIDS/STI Control Programme/Ghana Health Service; 2009.

[13] National AIDS/STI Control Programme/Ghana Health Service. HIV sentinel survey report 2016. Accra: National AIDS/STI Control Programme/Ghana Health Service; 2017.

[14] Ghana Health Service. National Yaws Control Programme: Annual report 2010. Accra: Ghana Health Service; 2011.

[15] Dassah ET, Adu-Sarkodie Y, Mayaud P (2016). Performance of syphilis sentinel surveillance in the context of endemic Treponematoses: experience from Ghana. BMC Infect Dis.

[16] Cochran WG (1977). Sampling Techniques.

[17] Dionne-Odom Jodie, Mbah Rahel, Rembert Nicole J., Tancho Samuel, Halle-Ekane Gregory E., Enah Comfort, Welty Thomas K., Tih Pius M., Tita Alan T. N. (2016). Hepatitis B, HIV, and Syphilis Seroprevalence in Pregnant Women and Blood Donors in Cameroon. Infectious Diseases in Obstetrics and Gynecology.

[18] Endris M, Deressa T, Belyhun Y, Moges F. Seroprevalence of syphilis and human immunodeficiency virus infections among pregnant women who attend the University of Gondar teaching hospital, Northwest Ethiopia: a cross sectional study. BMC Infect Dis 2015; 15:111. 10.1186/s12879-015-0848-5. Accessed 09 Nov 2018. 10.1186/s12879-015-0848-5 PMC4353477 25887081

[19] Mutagoma Mwumvaneza, Balisanga Helene, Remera Eric, Gupta Neil, Malamba Samuel S, Riedel David J, Nsanzimana Sabin (2016). Ten-year trends of syphilis in sero-surveillance of pregnant women in Rwanda and correlates of syphilis-HIV co-infection. International Journal of STD & AIDS.

[20] Amsalu A, Ferede G, Assegu D. High seroprevalence of syphilis infection among pregnant women in Yiregalem hospital southern Ethiopia. BMC Infect Dis 2018; 18:109. 10.1186/s12879-018-2998-8. Accessed 09 Nov 2018. 10.1186/s12879-018-2998-8 PMC5840736 29510664

[21] Kubio Chrysantus, Tierney Geraldine, Quaye Theophilus, Nabilisi James Wewoli, Ziemah Callistus, Zagbeeb Sr Mary, Shaw Sandra, Murphy William G. (2012). Blood transfusion practice in a rural hospital in Northern Ghana, Damongo, West Gonja District. Transfusion.

[22] Asiki G., Mpendo J., Abaasa A., Agaba C., Nanvubya A., Nielsen L., Seeley J., Kaleebu P., Grosskurth H., Kamali A. (2011). HIV and syphilis prevalence and associated risk factors among fishing communities of Lake Victoria, Uganda. Sexually Transmitted Infections.

[23] Sakala J, Chizuni N, Nzala S. A study on usefulness of a set of known risk factors in predicting maternal syphilis infections in three districts of Western Province, Zambia. Pan Afr Med J 2016; 24:75. 10.11604/pamj.2016.24.75.8425. Accessed 09 Nov 2018. 10.11604/pamj.2016.24.75.8425 PMC5031372 27703597

[24] Shimelis T, Lemma K, Ambachew H, Tadesse E. Syphilis among people with HIV infection in southern Ethiopia: sero-prevalence and risk factors. BMC Infect Dis 2015; 15:189. 10.1186/s12879-015-0919-7. Accessed 09 Nov 2018. 10.1186/s12879-015-0919-7 PMC4406018 25884178

[25] Tessema B, Yismaw G, Kassu A, Amsalu A, Mulu A, Emmrich F, Sack U (2010). Seroprevalence of HIV, HBV, HCV and syphilis infections among blood donors at Gondar University teaching hospital, Northwest Ethiopia: declining trends over a period of five years. BMC Infect Dis.

[26] Ginindza TG, Stefan CD, Tsoka-Gwegweni JM, Dlamini X, Jolly PE, Weiderpass E, Broutet N, Sartorius, B. Prevalence and risk factors associated with sexually transmitted infections (STIs) among women of reproductive age in Swaziland. Infect Agents Cancer 2017; 12 Suppl 1: 29. 10.1186/s13027-017-0140-y. Accessed 15 Nov 2018. 10.1186/s13027-017-0140-y PMC5445272 28559923

[27] Manda Samuel O.M., Lombard Carl J., Mosala Thabang (2012). Divergent spatial patterns in the prevalence of the human immunodeficiency virus (HIV) and syphilis in South African pregnant women. Geospatial health.

[28] Kizito Dennison, Woodburn Patrick W., Kesande Beleth, Ameke Christine, Nabulime Juliet, Muwanga Moses, Grosskurth Heiner, Elliott Alison M. (2008). Uptake of HIV and syphilis testing of pregnant women and their male partners in a programme for prevention of mother-to-child HIV transmission in Uganda. Tropical Medicine & International Health.

[29] Osei-Yeboah James, Lokpo Sylvester Yao, Ussher Francis Abeku, Orish Verner Ndudiri, Hamid Abdul-Wahab Mawuko, Dakorah Mavis Puopelle, Ntoni Tibemponi, Nani Emmanuel Agbeko, Ayroe Felix, Adigbli Daniel (2018). The Epidemiology of Human Immunodeficiency Virus (HIV) and Syphilis in Ghana: A Five-Year Single Urban Site Parallel Population-Based Analysis vis-à-vis the Sentinel Survey. Journal of Tropical Medicine.

[30] Urassa W, Moshiro C, Chalamilla G, Mhalu F, Sandstrom E. Risky sexual practices among youth attending a sexually transmitted infection clinic in Dar es Salaam, Tanzania. BMC Infect Dis. 2008;8:159. 10.1186/1471-2334-8-159. Accessed 09 Nov 2018. 10.1186/1471-2334-8-159 PMC2596153 19019224

[31] Kumogola Y, Slaymaker E, Zaba B, Mngara J, Isingo R, Changalucha J, Mwidunda P, Kimaro D, Urassa M (2010). Trends in HIV & syphilis prevalence and correlates of HIV infection: results from cross-sectional surveys among women attending ante-natal clinics in Northern Tanzania. BMC Public Health..

[32] Nankinga O, Misinde C, Kwagala B. Gender relations, sexual behaviour, and risk of contracting sexually transmitted infections among women in union in Uganda. BMC Public Health. 2016;16:440. 10.1186/s12889-016-3103-0. Accessed 09 Nov 2018. 10.1186/s12889-016-3103-0 PMC4881206 27229928

[33] SA ZHIHONG, LARSEN ULLA (2008). GENDER INEQUALITY INCREASES WOMEN’S RISK OF HIV INFECTION IN MOSHI, TANZANIA. Journal of Biosocial Science.

[34] Kazadi WM, Asiedu KB, Agana N, Mitjà O. Epidemiology of yaws: an update. Clin Epidemiol. 2014;6:119–28. 10.2147/CLEP.S44553. Accessed 28 Feb 2019. PMC3979691 24729728

[35] Ghinai Rosanna, El-Duah Philip, Chi Kai-Hua, Pillay Allan, Solomon Anthony W., Bailey Robin L., Agana Nsiire, Mabey David C. W., Chen Cheng-Yen, Adu-Sarkodie Yaw, Marks Michael (2015). A Cross-Sectional Study of ‘Yaws’ in Districts of Ghana Which Have Previously Undertaken Azithromycin Mass Drug Administration for Trachoma Control. PLOS Neglected Tropical Diseases.

